# Can deep learning classify stroke subtypes from chest X-rays?

**DOI:** 10.1016/j.ebiom.2021.103517

**Published:** 2021-08-04

**Authors:** Vineet K. Raghu, Michael T. Lu

**Affiliations:** Massachusetts General Hospital Cardiovascular Imaging Research Center and Harvard Medical School, Boston, MA, United States

Deep neural networks have been shown to diagnose and predict risk of disease based on medical imaging [1]. Chest radiographs (x-ray or CXR) present a tremendous opportunity for deep learning algorithms. They are one of the most common tests in medicine and can be a window into systemic health and disease, especially for cardiovascular and respiratory systems. Most work in this field has been focused on using deep learning to mimic a radiologist's read of the CXR, akin to an automated written report [2]. More recently, researchers have explored the use of deep learning to accomplish objectives not currently performed by radiologists such as risk estimation [3,4] or complex diagnostic tasks [2]. In this article, Jeong and colleagues explore another interesting classification problem by testing whether a deep learning model (they call ASTRO-X) can classify cardioembolic from noncardioembolic stroke based on a single CXR image [5].

Cardioembolic strokes account for 14–30% of all cerebral infarcts, and their incidence is rising, possibly due to increased life expectancy and better prevention of other causes of stroke (e.g., improved treatment for hypertension and dyslipidemia) [6]. Cases of cardioembolic stroke are characterized by a major cardiac source of embolism without significant arterial disease [6]. Diagnosing cardioembolic stroke involves a combination of neuroimaging, cardiac imaging, and laboratory measures. For example, the TOAST (Trial of Org 10172 in Acute Stroke Treatment) system, which was used as the gold standard in this study, states that a diagnosis of cardioembolic stroke requires identifying at least one cardiac source of an embolus and eliminating the possibility of large-artery atherosclerosis as the cause of thrombosis/embolism [7].

In this work, the authors developed and tuned the ASTRO-X model using CXRs from 3,255 patients with acute ischemic stroke from a single institution. Patients with unknown or undetermined etiology of stroke were excluded (*N* = 2,175), and so the ASTRO-X model focuses on distinguishing cardioembolic from non-cardioembolic in those with known acute ischemic stroke. The final ASTRO-X model was evaluated in two testing sets: 809 different patients from the same institution used for training, and 750 patients from 7 different hospitals. The authors found that ASTRO-X discriminated cardioembolic stroke from other subtypes with high accuracy (AUC of 0.86 in the internal test set and 0.82 in the external) and had added value over a risk factor-based regression model (Combined AUC 0.89 vs. risk factors 0.83).

What information did ASTRO-X capture beyond already known risk factors in the regression models? Unfortunately, deep learning models are black boxes, so it is impossible to say with certainty. The authors used Gradient-weighted class activation mapping (Grad-CAM heatmaps), which highlight regions of the image that the deep learning model is primarily using to make a prediction. They found that these heatmaps focused on the left atrium, a reasonable place to detect anatomical changes caused by chronic atrial fibrillation. Indeed, atrial fibrillation was substantially more common in the cardioembolic stroke population (76%) than the non-cardioembolic patients (0.6%). However, in 43% of the cardioembolic cohort with atrial fibrillation, atrial fibrillation was not yet diagnosed at the time of admission. The addition of ASTRO-X predictions to a multivariable model including a known diagnosis of atrial fibrillation at admission resulted in a significant increase in AUC for distinguishing cardioembolic from non-cardioembolic stroke. Associations with echocardiographic findings further validated that ASTRO-X is associated with changes to left atrial and ventricular function. Despite the known limitations of heatmap-based approaches (e.g., low-resolution and over-interpretation, as non-highlighted parts may still contribute substantially to predictions), the association with echocardiographic findings improves confidence in the results.

A major open question is whether ASTRO-X can impact clinical care of acute stroke. The authors suggest that ASTRO-X can help by “reducing human errors or guiding more thorough work-up for cardiac problems based on the probability”. But how would the results be presented? One possibility is to suggest cardioembolic stroke automatically when an ischemic stroke diagnosis and suggestive ASTRO-X score are entered into the medical record. Another is to present the probabilities at the request of the attending physician when they are uncertain. However, if thorough neuroimaging, cardiac imaging, and atrial fibrillation workup is necessary to make a final classification regardless, what impact would the suggestion of the deep learning model have on final patient care? Would the advice of ASTRO-X be considered in the diagnosis? These are all questions that the AI community is dealing with for many newly developed deep learning models [8].

Overall, the study is an interesting approach to improve the classification and management of patients with acute ischemic stroke. It adds to the growing body of literature showing that deep learning applied to ubiquitously available medical imaging can substantially improve upon existing methods to diagnose, prognose, and predict risk of disease.

## Declaration of Competing Interest

Dr. Lu is supported by 10.13039/100000968American Heart Association grants 18UNPG34030172 and 810966; has common stock in Nvidia and Advanced Micro Devices; reports research funding as a co-investigator to Massachusetts General Hospital from Kowa Company Limited and Medimmune/AstraZeneca; and received personal fees from PQBypass unrelated to this work. Dr. Raghu has common stock in Nvidia, Alphabet, and Apple and is supported by 10.13039/100009645National Academy of MedicineHealthy Longevity Grand Challenge (2000011734) and NIH grant T32HL076136.
